# Cases and Reflections on the Use of the Gum Elastic Air Bag

**Published:** 1860-04

**Authors:** W. H. Byford

**Affiliations:** Professor of Obstetrics, etc., in the Medical Department of Lind University


### CASES AND REFLECTIONS ON THE USE OF THE GUM ELASTIC AIR BAG.

By W. H. BYFORD, A. M., M. D.,

Professor of Obstetrics, etc., in the Medical Department of Lind University.

I was called to see a patient in consultation with Dr. P., who was suffering with symptoms of impacted pelvis; caused by retroversion of the uterus, viz: retention of urine and fœces, tenesmus of very distressing character, vomiting, irritative fever, great restlessness, etc. Her symptoms had been gradually increasing for three weeks, until they had become intolerable to her, and alarming to her friends and medical attendant. Two unsuccessful attempts had been made to replace the uterus. I found the pelvis literally crammed with that organ, pregnant nearly four months. There was hardly room to pass the finger up behind the symphisis pubis, and a little to one side. She complained of excruciating pain upon every touch, and declared she could not bear any manipulation for the rectification of the displaced organ.

We could not induce her to take a favorable position on the bed, and I operated while she was sitting on the lap of a female friend. I insinuated my fingers along the perineum, coccyx and hollow of the sacrum; gently, but firmly pushing the tumid uterus upwards and backward along the curve of the sacrum far as I could reach. Holding it in this position, I introduced my colpeuryuter, empty, as far up along the sacrum as I could make it go, and held it there until Dr. P. inflated it. Air was driven into it until it was the size of a large hen egg. I then pressed it thus inflated up high as I could reach, and again had more air thrown into it, until it had increased to nearly double the size after first inflation. Pressing it still upward and backward firmly against the upper part of the sacrum, the fundus suddenly slipped above the promontory, and placed itself into the natural position. I could then pass my fingers freely across the pelvis in any direction, and feel the os uteri occupying its centre.

The whole operation did not last longer than ten minutes, apparently was not attended by any increase of suffering, and was not succeeded by any bad symptoms. The patient expressed the relief she felt in the strongest terms immediately after the operation, and passed a large quantity of urine. She carried her child to full term, and did very well in every respect.

I prefer the elastic bag, as an extension to my fingers, to any solid instrument, because its soft airy elasticity defends the uterus from contusion, and in this case it proved very effective. I think this manner of using it very much better than passing it into the rectum—as recommended by some authors—and then inflating it. The painful distension of the rectum by the expansion of the instrument, I should think would be horrible, if carried to such an extent as to lift the uterus above the pelvic brim, and I think the bag would operate to less advantage than if properly managed in the vagina. In using the elastic bag, it should be moved high as possible along the hollow of the sacrum, with the fingers first, and then the bag, *empty*, placed well against the sacrum and up against the fundus uteri, and carried up, pushing the uterus before it while it is being inflated. It should not be inflated larger than merely to afford an extension for the fingers, and not so as to anywhere nearly fill the pelvis. We should then move it upward and backward, inflating and raising at the same time, until the fundus uteri rises above the promontory of the sacrum. The air should then be allowed to escape, and our instrument withdrawn. Too great an inflation will fill up the pelvis too much, and the impinging points of this medium of power will be too numerous and diffuse to be efficient, as our force should be as near the anterior surface of the sacrum as possible. When not bound down by adhesions, or it has not already acquired too great a size to raise through the superior straight, this mode of operating need not fail to replace a retroverted uterus.

A good and sufficient substitute when the elastic bag could not be procured, is a strong beef's or hog's bladder. By tying a small reed, a foot or eighteen inches long, into the urethal canal we could inflate this natural bag sufficiently for use while in the vagina.

The gum elastic bag is being used for many purposes about the pelvis. It makes the best tampon for hemorrhage in cases of abortions in early pregnancy, and placenta previa in the later stages of this condition. While checking the hemorrhage in these cases it excites the uterus to contraction. In consequence of this last effect it is better not to use it where there is any hope of saving the fœtus from expulsion. It acts admirably as a pessary in prolapsus uteri, where this affection is so bad as to be unmanageable by any other sort of mechanical contrivance. An instance of this kind occurred not long since in the practice of a friend of mine—a man of forty years experience in the profession—in which the uterus, as I witnessed myself, was expelled beyond the vulva, making a tumor four inches long, the os being the most dependent part.

Every kind of pessary and other device which could be thought of by any friend and others with whom he consulted, failed to retain the organ in the pelvis more than a few moments at a time, soon as she raised herself up and walked about, the instrument and uterus came tumbling out together. After returning the uterus, the colpeuryuter was introduced high up, and inflated till it produced slight uneasiness from distension. This retained the uterus in place for several hours, but so lax were the external soft parts that it also escaped. She now wears it with great comfort to herself by using a T bandage over the vulva. This bandage could not be made to retain any other kind of pessary in place, which plainly showed the advantage of this.

I see since the above was written some interesting observations in the last number of Braithewaite's retrospect, in respect to the use of the air-bag. From it, and observations of others, we are justified in using the bag as a plug in hemorrhage from the nose, anus, etc. Prof. Byford in Chicago Med. Exam[illegible]

